# Unusual sight-threatening complication of scaled burn injury in a pediatric patient: A case report

**DOI:** 10.1016/j.ijscr.2024.109700

**Published:** 2024-04-26

**Authors:** Anteneh Meaza Dawit

**Affiliations:** Addis Ababa University, College of Health Sciences, Department of Plastic and Reconstructive Surgery

**Keywords:** Ophthalmic complication, Endopthalmitis, Burn injury

## Abstract

**Introduction:**

Acute ophthalmic complications in burn injury patients are rare. Despite their rarity however, when they do occur, they may result in permanent visual impairment and lifelong disability. Emphasizing the need for vigilance and prompt treatment initiation for a good outcome.

**Case presentation:**

A 3-year-old girl was referred to our burn unit after receiving 10 days of treatment at a peripheral hospital for scalding burn to her upper chest, face, and scalp. Remarkably, her eyelids were spared from the initial injury. Upon her third day under our care, she began displaying severe sensitivity to light, photophobia, irritability, excessive crying, and frequent itching of the eyes. Upon examination, bilateral conjunctival redness and inflammation, as well as whitish opacity of both corneas with pus in the anterior chambers were observed. Prompt consultation and treatment led to improvement of the symptoms.

**Discussion:**

This case report describes a rare case of bilateral endopthalmitis in a burn injury patient with no initial apparent injury to the eyes and emphasizes the importance of vigilance, prompt recognition of the scenario and treatment initiation. Risk factors identified in this patient included compromised immune system due to severe burn, prolonged hospitalization, IV antibiotic use and young age. Other more common risk factors associated with endopthalmitis like direct ocular injury and central line use were absent making the case unusual.

**Conclusion:**

Despite their rarity severe ophthalmic complications like endopthalmitis can occur in burn injury patients even when least expected. Heightened vigilance, prompt evaluation, multidisciplinary team approach and early initiation of broad spectrum antibiotic treatment is crucial to prevent permanent visual loss and lifelong disability.

## Highlights

• This report describes a rare case of bilateral endopthalmitis that occured in a pediatric patient who sustained burn injury.
• Endopthalmitis as a rare ophthalmic complication of burn injury is entertained as an entity and implied as a complication of acute burn injuries.
• Vigilance, prompt recognition of the scenario and treatment initiation is emphasized.

## 1 Introduction

Acute ophthalmic complications following burn injuries are infrequent and the majority follow direct chemical injuries to the cornea and conjunctiva. Furthermore, thermal injury to the eyeball itself is rare due to the protective blink reflex.

Despite their rarity, ophthalmic complications when they occur in burn injury patients can have profound and lasting consequences. Their potential for lifelong disability necessitates early recognition and prompt initiation of treatment, even in scenarios where such complications might be least expected.

In this report, I present unusual ophthalmic complication that followed a scalding burn injury to the scalp and face, despite having no initial apparent injury to the eyes. Early recognition and prompt treatment led to the recovery of sight and improvement of symptoms. This underscores the importance of heightened vigilance even in scenarios where ophthalmic complications are least expected. Prompt evaluation, multidisciplinary team approach, and early treatment in the aftermath of burn injuries are emphasized. The rarity of this ophthalmic complication following burn injuries also warrants further studies into its pathophysiology. This report adheres to the SCARE criteria [1].

## 2 Case presentation

A 3-year-old female toddler was brought to our burn unit after sustaining a scalding burn injury to her upper trunk, face and scalp from a hot stew 10 days back. Initially the injury resulted in admission to a primary care hospital. During her stay at the primary hospital wound care, analgesia, and IV ceftriaxone were administered. On the 10th day, she was referred to our center for surgical treatment.

**Fig. 1:**
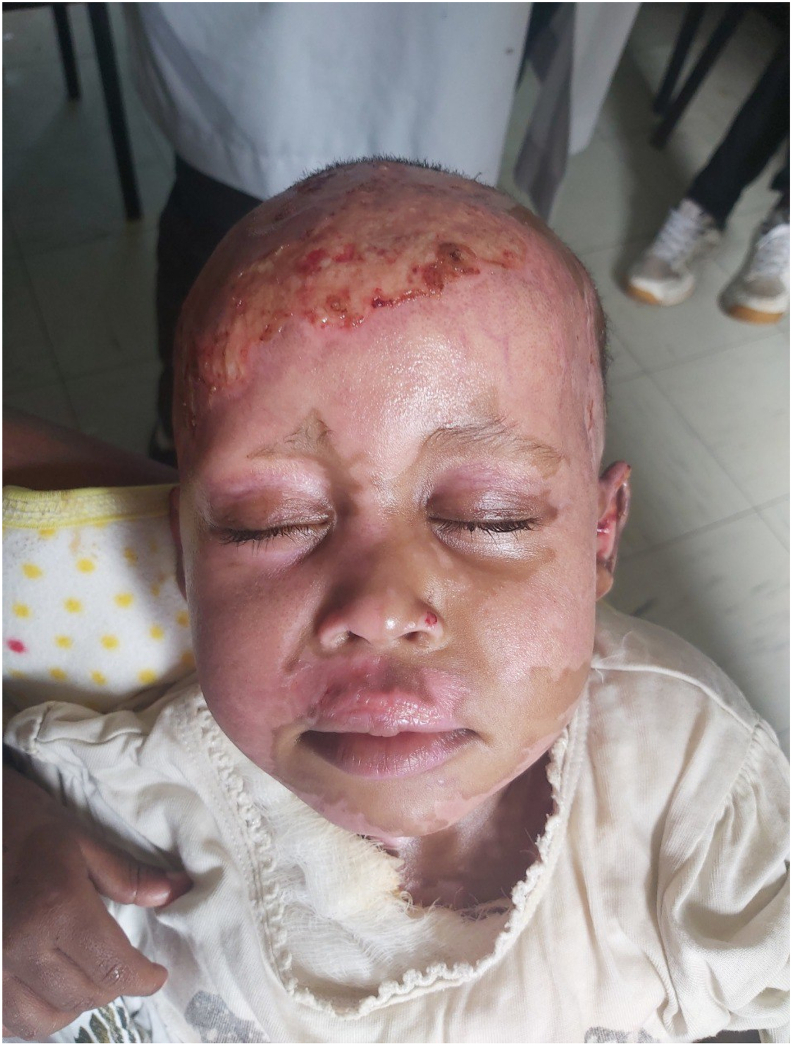
Shows post burn wound over the scalp and healed superficial burns over the cheeks and forehead. There is also sparing of the eyelids and eyelashes.

Upon presentation the patient exhibited stable vital signs. PR = 108, RR = 24, Temp = 36.4 °C, Physical findings included a mixed superficial and deep partial-thickness burn wound over the scalp, forehead, cheeks, neck, and shoulder. Importantly, the eyelids and eyelashes were spared, allowing proper eye lid closure (Fig. 1). The conjunctiva and sclera were normal.

Base line investigations: Showed normal parameters beside leukocytosis (Table 1).

**Table 1:** Baseline investigations done on presentation.

CBC	OFT	Electrolytes
WBC = 20.48 (N-64.9 %; L-23 %)	ALT = 45,	Na+ = 130 U/L
Hb = 9.5 g/dL, HCT = 27 %	AST = 52	K+ = 3.6 U/L
MCV = 78.7 fL, MCH = 27.6 pg	SCr = 0.7 mg/dL	
PLT = 274,000		

Subsequently the patient was admitted to the pediatric burn ward and management was started with daily wound care, dressing changes, analgesia and feeding.

**Fig. 2:**
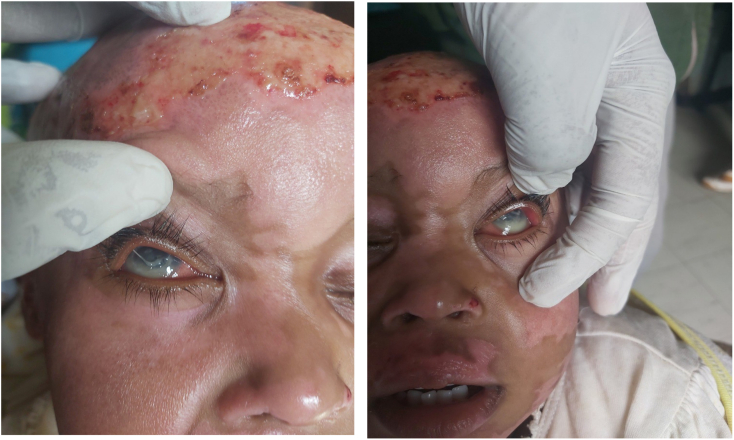
Right and left eye with hypopyon, conjunctival chemosis and injection.

**Fig. 3:**
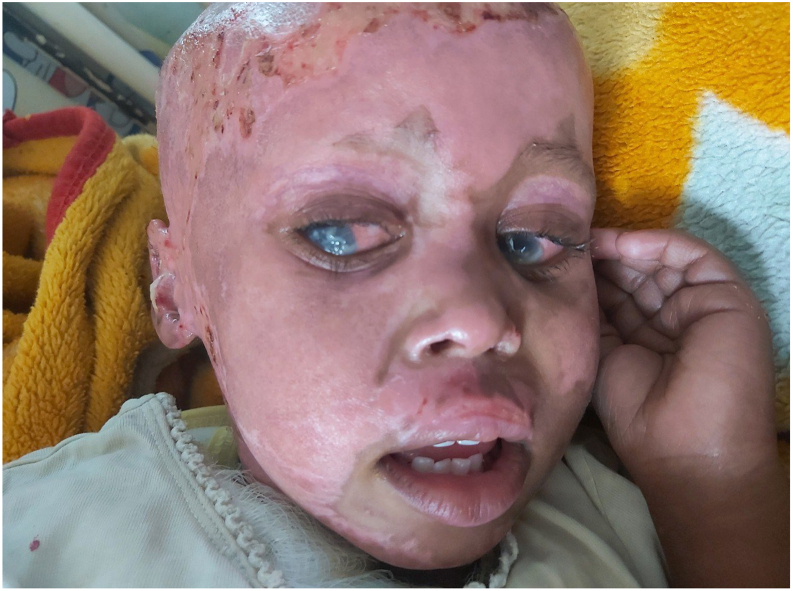
Bilateral hypopyon and conjunctival injection seen.

**Fig. 4:**
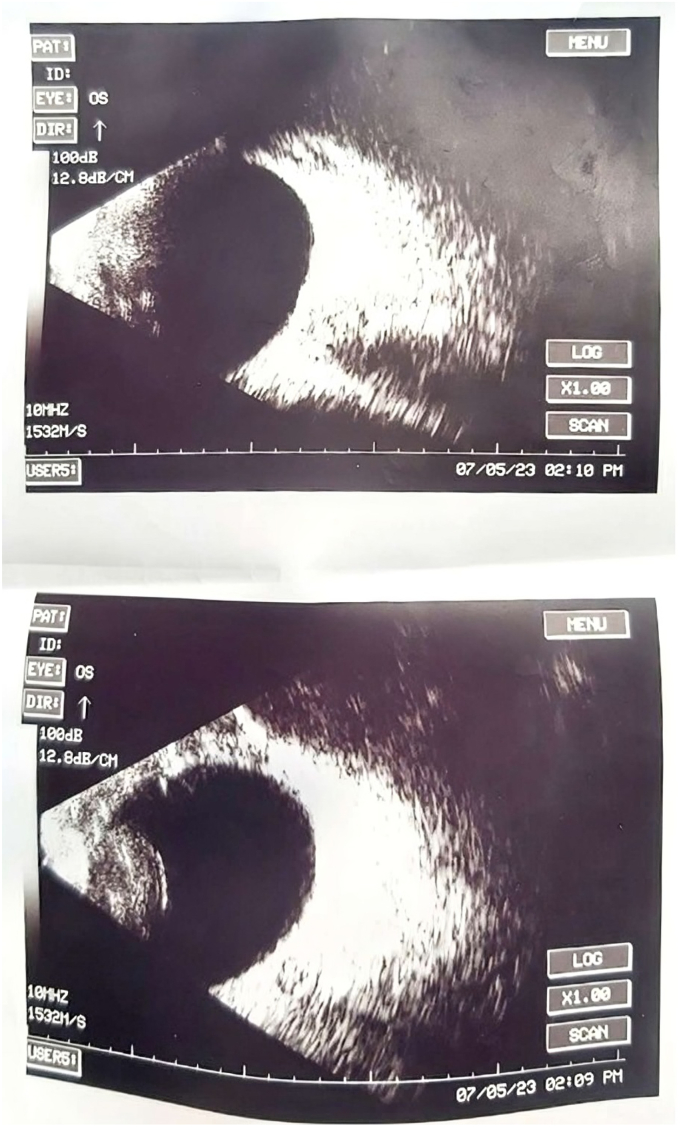
Ocular ultrasound - edematous lenses and minimal debris in the vitreous humor.

On her third day of admission she began to exhibit photophobia with aversion to light and irritability, excessive crying and frequent eye itching was as reported. Physical examination revealed bilateral conjunctival erythema and injection. Along with bilateral whitish opacity involving the bilateral cornea and pus accumulation in the anterior chambers (Fig. 2, Fig. 3). An orbit ultrasound was performed (Fig. 4), confirming a diagnosis of bilateral endophthalmitis. Culture couldn't be obtained due to limited resources in our setup. Urgent ophthalmologist consultation settled the diagnosis of post-traumatic endophthalmitis/OU.

**Fig. 5:**
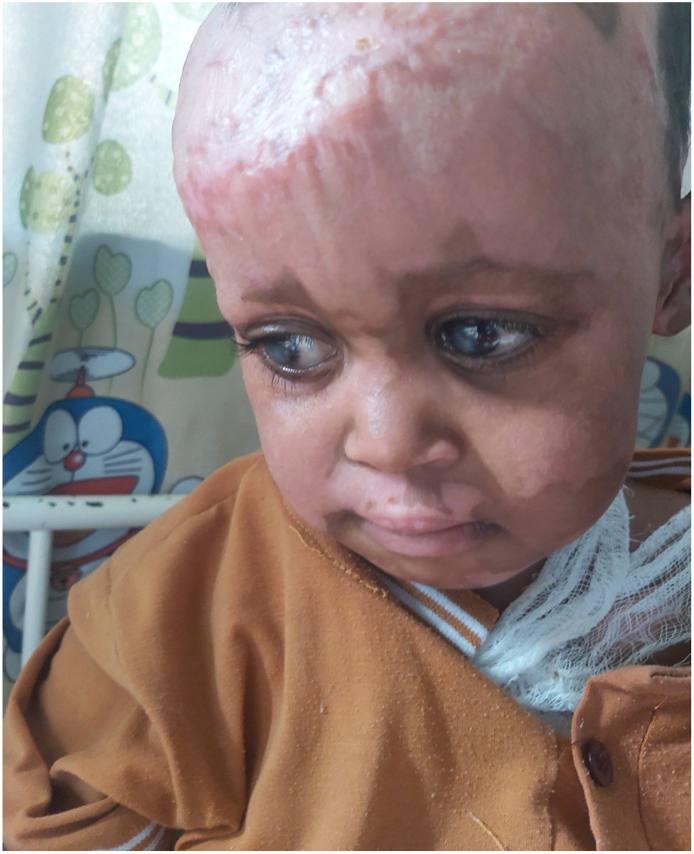
Appearance after treatment shows improvement. There is also minimal corneal opacity seen.

Prompt treatment was initiated with empirical broad spectrum antibiotics. Intravitral injection of Ceftazidime and Vancomycin was given for 2 rounds. Ceftazidime and Vancomycin eye drop QID and TTC eye ointment QID was continued for 10 days. The treatment led to marked improvement all of the symptoms and regain of her vision except mild corneal opacity (Fig. 5). The patient was later discharged after getting split thickness skin graft for the unhealed part of the burn wound over the anterior chest and is continuing follow up with ophthalmologist for the corneal scarring.

## 3 Discussion

This report describes a rare case of bilateral endopthalmitis in a pediatric burn injury patient with no initial apparent involvement of the eyes and emphasizes the importance of vigilance, prompt recognition the scenario and treatment even in cases where the occurrence is least suspected.

The risk factors for endopthalmitis identified in this patient include compromised immune system due to severe burn, prolonged hospitalization, IV antibiotic use and young age. Other more common risk factors like direct ocular injury or eyelid injury, sepsis and central line use were not present in this patient making the case unusual.

Ocular burn injuries usually occur from exposure to direct flame, scalding liquid, blast injury. Direct thermal injury to the globe is uncommon because of the blinking reflex and Bell's phenomenon (eyes rolling upward) which protect the ocular surface. Periorbital burns in contrast are the most common type of ocular injury, account for 48 % of cases [2]. Periorbital burns when present can predispose to eyelid contracture, lagophthalmos, exposure keratitis, corneal ulceration, perforation, endopthalmitis and blindness [2]. This patient had no initial apparent injury to the eyelids nor eyes that could have predisposed her for endopthalmitis (Fig. 1).

Endophthalmitis refers to intraocular inflammation secondary to infection, usually bacterial or fungal. It is potentially devastating and carries a poor visual prognosis in most patients. It is classified as exogenous or endogenous depending on the route of infection to the eye [3]. Exogenous endophthalmitis occurs when microorganisms enter the eye from a breach of external ocular barriers (e.g., intraocular surgery/injections or penetrating trauma) [3].

Contrastingly, endogenous endophthalmitis occurs when microorganisms enter the eye by crossing the blood-ocular barrier via hematogenous spread from a distant source of infection. In these cases, there is no direct trauma to the eye, and as such, eye protection would not protect against this condition. Endogenous endophthalmitis is less common than the exogenous form and is responsible for about 2–8 % of endophthalmitis cases [3]. Both are ophthalmological emergencies and often pose a diagnostic challenge since the presentation can be extremely heterogenous [3,4].

Endogenous bacterial endophthalmitis (EBE) in burns patients is rare. There are currently only 2 case reports that have described patients with significant burns of >40 % TBSA [3]. Patients with EBE usually report symptoms of blurred vision, floaters, eye redness, eye pain, and photophobia which were present in this patient. Involvement may be bilateral in 20 % of patients [3] which is also seen in this patient.

Eye findings and their evolution can vary markedly. Fulminating infection may present with rapid visual loss, hypopyon, uveitis, and dense vitritis obscuring any fundus view which was seen in this patient. In this case, progression to orbital cellulitis may follow with proptosis and restriction of eye movements [3]. Low-grade or indolent presentations may cause relatively mild anterior uveitis and vitritis with fundus lesions, such as Roth spots (white-centered retinal hemorrhages), retinal vasculitis, or sub retinal abscesses [3].

Following suspicion, a thorough evaluation should follow. Microbiological investigations are key to confirming diagnosis [3]. In this patient microbiologic investigations were not done due to lack of capable facility. In addition, as long as there is ample clinical ground for the diagnosis culture should not be mandatory for all patients.

Once clinically suspected endopthalmitis requires prompt and high dose broad spectrum systemic antibiotic treatment. Waiting for culture results should not delay antibiotic initiation and the role of intravitral antibiotics and vitrectomy surgery is less clear [3]. Systemic antifungal agents should also be considered in addition to broad spectrum antibiotic coverage in setups that lack capabilities for culture [5]. Ophthalmologist involvement, broad spectrum antibiotic initiation, injections and close follow-up were crucial in this patient's good outcome.

The more severe cases of endopthalmitis might end up in evisceration due to complete blindness and intractable pain. The visual outcome of EBE is also poor, with 32–41 % of patients achieving counting fingers vision or better; 26–57 % losing their vision, and 14–29 % requiring evisceration or enucleation. EBE also has mortality rate of 5 % from the consequences of sepsis [3]. Early diagnosis and prompt treatment may improve these outcomes [3,4] as in this case.

## 4 Conclusion

In conclusion, despite their rarity severe ophthalmic complications like endopthalmitis might occur in burn injury patients even when their occurrence is least expected. Heightened vigilance, prompt evaluation, multidisciplinary team approach and early initiation of broad spectrum treatment is crucial to prevent permanent visual loss and lifelong disability. The rarity of bilateral endopthalmitis as a clinical entity in burn injury patients also warrants further research on the topic and this case report can be used as a valuable supplement for further studies.

## Consent

Written informed consent was obtained from the patient's parents/legal guardian for publication and any accompanying images. A copy of the written consent is available for review by the Editor-in-Chief of this journal on request.

## Ethical approval

No ethical approval was needed for a case report writing in my institution.

## Funding

This report did not receive any form of grant from funding agencies in the public, commercial, or not-for-profit sectors.

## Guarantor

The author will take full responsibility for the work.

## CRediT authorship contribution statement

The author was responsible for treating the patient and writing the paper.

## Declaration of competing interest

No conflict of interest has affected this report.
